# Acute Cardiac Manifestations of SARS-CoV-2 Infection: Spotting the Clot

**DOI:** 10.1155/2023/6366959

**Published:** 2023-09-14

**Authors:** Robert N. Kerley, Amal John, Sajjad Matiullah, Mark Rogan

**Affiliations:** ^1^Department of Medicine, University College Cork, Ireland; ^2^University Hospital Waterford, Dunmore Road, Waterford, Ireland

## Abstract

A middle-aged gentleman presented with a one-week history of progressive dyspnoea on minimal exertion, persistent haemoptysis, and right calf swelling. His only past medical history of note was a recently positive SARS-CoV-2 nasopharyngeal swab performed as part of a workplace outbreak screening. A CT pulmonary angiogram (CTPA) showed bilateral pulmonary thrombi, extensive consolidation, and a left ventricular (LV) thrombus. A transthoracic echocardiogram (TTE) showed a dilated LV with severely impaired systolic function and LV thrombus. The patient was anticoagulated with warfarin, commenced on IV diuretics and COVID-19 protocol. Cardiac magnetic resonance (CMR) imaging showed a severely dilated nonischaemic cardiomyopathy with a heavy thrombus burden and a fibrosis pattern in keeping with myocarditis. We present a case of COVID-19-related myocardial dysfunction with high thrombotic burden and a discussion of its management.

## 1. Background

Clinicians must be aware of the cardiac manifestations of SARS-CoV-2 infection which can include but are not limited to myocarditis. The main complications of SARS-CoV-2 infection include pneumonia, acute respiratory distress syndrome (ARDS), pneumothorax, myocardial injury, and thromboembolic complications including venous thromboembolism (VTE) and pulmonary thrombosis [1]. In terms of cardiovascular complications, patients are often asymptomatic with evidence of cardiac disease on cardiac investigations without symptoms of heart disease. This may progress to myocardial injury, myocarditis, stress cardiomyopathy, myocardial infarction, heart failure, or even cardiac arrhythmia, the most common of which is sinus tachycardia [2]. Specifically, this case broaches the management of heart failure, myocarditis, arrhythmia, and thrombotic events in a patient with concomitant interstitial pneumonia and ARDS. The role of cardiac MRI (CMR) to capture typical patterns of myocardial involvement will be highlighted with a discussion of the main differential diagnoses to consider. Overall, the educational aim of this case is to highlight the cardiac complications of SARS-CoV-2 with an emphasis on the investigations involved in their diagnosis and the therapeutic challenge they present.

## 2. Case Presentation

A middle-aged gentleman was referred to our Emergency Department (ED) with a one-week history of progressive dyspnoea on minimal exertion, persistent haemoptysis on a background of recent SARS-CoV-2 viral illness. He noted a one-month history of right calf swelling prior to developing dyspnoea. He had no background medical history of note and did not take any regular medications. His SARS-CoV-2 illness was mild and unremarkable having been diagnosed as part of a workplace outbreak screening.

On physical examination, the patient was tachycardic (110 beats/min) and tachypnoeic (28 breaths/min) with a stable blood pressure (121/63 mm Hg) and oxygen saturations of 90% on room air. On auscultation, inspiratory crepitations were noted at both lung bases and jugular venous pressure was elevated 5 cm above the angle of Louis. There was no evidence of a third heart sound or murmurs on auscultation. There was evidence of bilateral lower limb swelling with a 3 cm difference between the two calf muscles with the right measuring larger than the left.

### 2.1. Investigations

**Figure 1:**
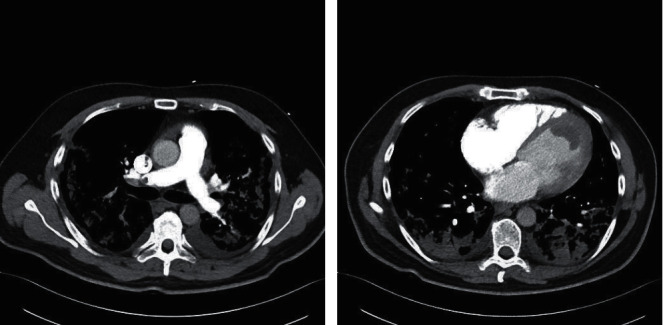
CT pulmonary angiogram. (a) Evidence of pulmonary thrombi at the bifurcations of the right main pulmonary artery and (b) cardiomegaly with an LV filling defect suggestive of a thrombus and multiple areas of consolidation consistent with SARS-CoV-2 infection.

**Figure 2:**
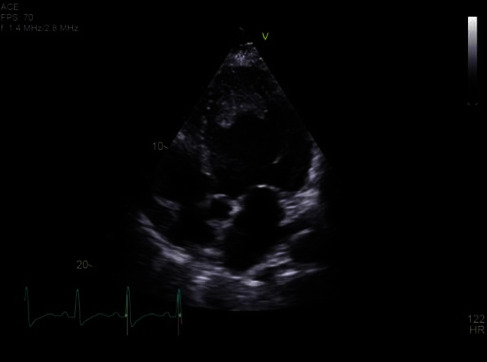
Transthoracic echocardiogram showed globally impaired left ventricular systolic function and a large left ventricular thrombus.

An electrocardiogram (ECG) was performed which showed sinus tachycardia (110 beats/min) and a narrow QRS complex without evidence of ischaemic changes. Admission blood profile showed a significantly elevated D-dimer (23.27 mg/L), marginally elevated troponin T (16.1 ng/L), and elevated pro-BNP (6360 pg/mL). Arterial blood gas showed type 1 respiratory failure. Inflammatory markers were significantly elevated with a leucocytosis (18.4 × 10^9^/L), neutrophilia (16.06 × 10^9^/L), hyperferritinaemia (4176 *μ*g/L), and a raised CRP (338.8 mg/L). His lymphocytes were normal throughout admission. His renal function and electrolytes were normal. His liver function tests were marginally deranged (ALT 97 IU/L, ALP 141 IU/L, and GGT 138 IU/L) with a normal total bilirubin (20.5 *μ*mol/L) and decreased synthetic function (INR 1.2, albumin 24 g/L). A chest X-ray showed extensive bilateral peripheral predominant airspace opacification. The patient proceeded to CT pulmonary angiogram (CTPA) and bedside transthoracic echocardiogram (TTE) as shown in Figures 1 and 2, respectively. CTPA showed pulmonary thrombi at the bifurcations of the right main artery, cardiomegaly with an LV filling defect suggestive of thrombus, and multiple areas of consolidation consistent with extensive COVID-19 infection (Figures 1(a) and 1(b)). A TTE showed globally impaired LV systolic function with global hypokinesis and an ejection fraction of 10-15% by Simpson's biplane method. The LV was dilated with an LV end diastolic volume of 254 mL and an LV internal diameter in diastole of 6.1 cm. A large LV mass was found at the LV apex with global severe hypokinesis (Figure 2). The mass was homogenously echogenic, protruding into the LV, and given the clinical context, this was presumed to be an LV thrombus, but a cardiac MRI (CMR) was ordered to further elucidate this. There was no evidence of right ventricular strain on ECG, TTE, or CTPA. The inferior vena cava was dilated at 2.3 cm with less than 50% respiratory variability. The patient's admission nasopharyngeal swab was negative but given concerning investigations was placed on our hospital COVID-19 protocol. His repeat swab was positive for COVID-19 infection.

### 2.2. Differential Diagnosis and Treatment

Following initial investigations, our working diagnosis was COVID-19 pneumonia with a high thrombotic burden in the form of extensive pulmonary thrombi, lower limb deep venous thrombosis, and LV thrombus with a possible diagnosis of SARS-CoV-2-induced myocarditis.

In terms of COVID-19 management, our patient was commenced on our hospital protocol of IV ceftriaxone 2 g once daily, dexamethasone 6 mg once daily (for ten days), five days of IV remdesivir (200 mg on the first day, four subsequent days of 100 mg per day), and ivermectin 400 mcg/kg once daily (for five days). It should be noted that ivermectin is no longer recommended in the treatment of COVID-19, but this was our hospital protocol prior to the publication of said data [3]. Our patient's oxygen requirement was minimal only requiring 2 litres of oxygen delivered via nasal prongs at a maximum.

In terms of thrombotic burden, therapeutic anticoagulation with low molecular weight heparin was commenced in the ED overnight. The following morning in conjunction between cardiology, respiratory medicine, and haematology, a decision was made to anticoagulate with a vitamin K antagonist given its superior evidence base in treating LV thrombi. Bridging with enoxaparin 1 mg/kg twice daily until the patient's INR was greater than 2.5 was employed.

**Figure 3:**
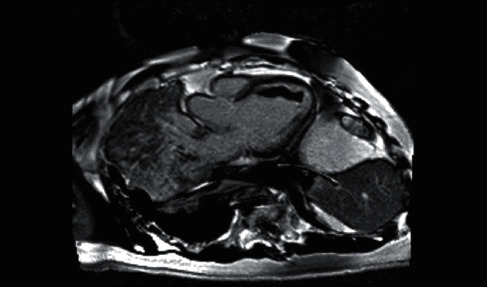
Cardiac resonance imaging showed a severely dilated left ventricle with severely impaired systolic function and late gadolinium enhancement in a subepicardial distribution.

In terms of heart failure, blood pressure became labile in response to bolus dose diuretics and a continuous IV furosemide infusion 120 mg/day was commenced which was tolerated over three days of diuresis. Liver function derangement resolved in response to diuresis. There was difficulty introducing LV remodelling medications due to hypotension and presyncope. Rate controlling agent bisoprolol 1.25 mg was started and uptitrated to 7.5 mg with the addition of ivabradine 5 mg due to ongoing tachycardia. Ramipril 1.25 mg and eplerenone 12.5 mg were introduced but could not be uptitrated due to intolerance. The aetiology of the patient's cardiomyopathy was investigated with an unremarkable coronary angiogram, thyroid function tests, autoimmune profile, HbA1c, and serum protein electrophoresis. CMR imaging was performed which showed a severely dilated LV (LV end diastolic volume 259 mL) with severe global impairment of LV systolic function (LV ejection fraction 19%) and late gadolinium enhancement in a subepicardial distribution of the inferolateral wall in keeping with myocarditis (Figure 3). There was no evidence of oedema on T2-STIR-weighted imaging. In addition, there was evidence of a heavy thrombotic burden as shown by the large LV thrombus and smaller RA thrombus on T1-weighted imaging. A hypercoagulability screen was conducted as advised by our haematology colleagues including protein C, protein S, antithrombin III, lupus anticoagulant, glycoprotein antibodies, anticardiolipin, factor V Leiden, and prothrombin gene mutations all of which were negative.

### 2.3. Outcome and Follow-Up

**Figure 4:**
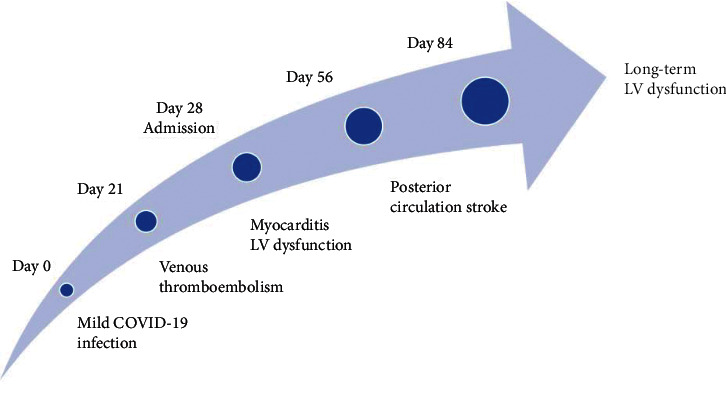
Case timeline.

Our patient had severe SARS-CoV-2-related illness complicated by pneumonia, deep vein thrombosis, pulmonary thrombi, intracardiac thrombi, and severely impaired LV function (Figure 4). The diagnosis of SARS-CoV-2-induced myocarditis was confirmed on CMR. Following four weeks of inpatient investigations and management, our patient was discharged on LV remodelling medications (ramipril 1.25 mg, bisoprolol 7.5 mg, eplerenone 12.5 mg, and ivabradine 5 mg). He was reviewed in clinic one week after discharge with a follow-up TTE performed in the interim with an LVEF of 20-25% and a small mobile LV thrombus which significantly reduced in size compared to previous. There was no evidence of RA thrombus on follow-up TTE. He returned to the clinic without concerning decompensatory symptoms but ongoing tachycardia with a pulse rate of 104 bpm. Cardiac examination was unremarkable, and he was clinically euvolemic. His INR remained therapeutic at 3.6 on warfarin 2 mg per day. With advice from haematology, he was planned for six months of anticoagulation to treat SARS-CoV-2 provoked pulmonary thrombi and intracardiac thrombi. Unfortunately, at 4 weeks postdiagnosis, he was admitted with an MRI-confirmed posterior circulation stroke due to labile INR readings. He was switched to dabigatran 150 mg BD in conjunction with the stroke and haematology team, and he was discharged without a functionally limiting neurological deficit. A reversible direct oral anticoagulant was chosen by our stroke team due to the risk of further stroke and haemorrhagic transformation with an LV thrombus.

## 3. Discussion

SARS-CoV-2 is proposed to gain entry into cardiomyocytes via the spike protein on angiotensin-converting enzyme 2 (ACE2). Multiple mechanisms have been proposed to explain this myocardial injury including (1) direct viral myocardial injury, (2) thrombosis in the myocardial microcirculation, (3) systemic inflammation, (4) acute coronary syndrome, and (5) myocardial injury secondary to hypoxaemia [1, 4].

The clinical presentation varies among reported cases, but the most emergent presentation is unfortunately fulminant myocarditis, defined as a sudden and severe inflammation of the myocardium resulting in ventricular dysfunction and heart failure within weeks of contracting the virus [5]. Bedside investigations such as serial ECG and cardiac biomarkers can raised suspicion of cardiac complications of SARS-CoV-2. Cardiac imaging with TTE and CMR can be used to distinguish between differential diagnoses. An invasive angiogram will often be required with concomitant endomyocardial biopsy (EMB) providing definitive diagnosis [6]. However, in the above patient's case, an EMB was not possible due to the presence of DVT/PE, coagulopathy, and an LV thrombus.

In a meta-analysis including 199 patients with SARS-CoV-2 infection, the most common CMR findings were active inflammation by T1 mapping (70%), oedema on T2/STIR (51%), late gadolinium enhancement (43%), pericardial effusion (24%), and pericardial involvement (22%) [7, 8]. The long-term clinical significance of CMR findings in COVID-19 is yet undetermined; however, the use of tissue characterization techniques including LGE sequences for myocardial necrosis and T2 mapping (or equivalent) sequences for myocardial oedema detection may provide strong prognostic value in the future.

Overall, clinical reasoning should drive the pursuit of definitive investigations when SARS-CoV-2-induced myocarditis is suspected.

The learning and take-home points for this case include the following:

- The main complications of SARS-CoV-2 infection include pneumonia, acute respiratory distress syndrome, pneumothorax, myocardial injury, and thromboembolic complications including venous thromboembolism and pulmonary thrombosis
- The clinical presentation of cardiovascular complications related to SARS-CoV-2 infection varies among reported cases, but the most emergent presentation is unfortunately fulminant myocarditis
- The most common CMR findings post-SARS-CoV-2 infection are active inflammation by T1 mapping (70%), oedema on T2/STIR (52%), late gadolinium enhancement (43%), pericardial effusion (24%), and pericardial involvement (22%)

## Data Availability

The underlying dataset underpinning this case report is available on request from the corresponding author.
